# Statistical shape models of cuboid, navicular and talus bones

**DOI:** 10.1186/s13047-016-0178-x

**Published:** 2017-01-31

**Authors:** Aleksandra U. Melinska, Patryk Romaszkiewicz, Justyna Wagel, Bartlomiej Antosik, Marek Sasiadek, D. Robert Iskander

**Affiliations:** 1 Department of Biomedical Engineering, Wroclaw University of Science and Technology, 50370, Wybrzeze Wyspianskiego, Wroclaw, Poland; 2 Regional Specialist Hospital, Research and Development Centre, Chair of Orthopaedics, Kamienskiego, Wroclaw, 24105 Poland; 3 Department of General Radiology, Interventional Radiology and Neuroradiology, Chair of Radiology, Wroclaw Medical University, Borowska, Wroclaw, 24105 Poland

**Keywords:** Bone model, Calcaneus, Cuboid, Navicular, Talus, Morphometry, Spherical harmonics, Shape modelling

## Abstract

**Background:**

The aim was to develop statistical shape models of the main human tarsal bones that would result in novel representations of cuboid, navicular and talus.

**Methods:**

Fifteen right and 15 left retrospectively collected computed tomography data sets from male individuals, aged from 17 to 63 years, with no known foot pathology were collected. Data were gathered from 30 different subjects. A process of model building includes image segmentation, unifying feature position, mathematical shape description and obtaining statistical shape geometry.

**Results:**

Orthogonal decomposition of bone shapes utilising spherical harmonics was employed providing means for unique parametric representation of each bone. Cross-validated classification results based on parametric spherical harmonics representation showed high sensitivity and high specificity greater than 0.98 for all considered bones.

**Conclusions:**

The statistical shape models of cuboid, navicular and talus created in this work correspond to anatomically accurate atlases that have not been previously considered. The study indicates high clinical potential of statistical shape modelling in the characterisation of tarsal bones. Those novel models can be applied in medical image analysis, orthopaedics and biomechanics in order to provide support for preoperative planning, better diagnosis or implant design.

**Electronic supplementary material:**

The online version of this article (doi:10.1186/s13047-016-0178-x) contains supplementary material, which is available to authorized users.

## Background

The statistical shape model (SSM) has been established as a powerful tool for medical image analysis [1–6]. The goal of constructing a statistical shape model is to obtain a mean shape and description of variation from a collection of samples [7–10]. The methods employed strongly depend on the chosen shape representation, which can be landmarks and meshes, medial models, Fourier surfaces, spherical harmonics, deformable models, wavelets description, non-uniform rational B-Splines and others [11, 12]. The choice of the shape representation influences further processing and calculation and in that context landmark-based point distribution models have become popular and commonly used methods. Statistical shape models are usually used for the task of segmentation [13, 14], but they could also be considered for finite element (FE) modelling [15, 16] and automatic detection of shape and feature correspondences [17, 18]. The SSM based techniques of medical image analysis have been applied to segmentation of bones [19–22] but only few studies considered statistical shape of the calcaneus, cuboid, navicular and talus that constitute the four largest tarsal bones [23–26]. There have been many studies considering modelling of foot bones. For example, Camancho et al. [27] generated an anatomically detailed, three-dimensional reconstruction of a human foot from computed tomography (CT) images. They proposed an accurate representation of bone and soft tissues of foot. The presented method became a base for further development of a FE model of the human foot that could be used in quantifying morphometric characteristics between different foot types [28]. Also, Liu et al. [29] described rigid model-based 3D segmentation of joints imaged using magnetic resonance (MR) and CT images in order to examine their kinematics. Of all tarsal bones, talus has received most of attention. Leardini et al. [30] proposed a geometric two-dimensional model of the ankle joint, which allows examining ankle stability. The presented model showed the path of calcaneus, ligament orientations, instantaneous axis of rotation, and conjugate talus surface profile as observed in the experiments. In their following work, [31] they aimed at developing a model of the intact human ankle complex. The goal was to design the total ankle replacement which would better reproduce the physiological function of the joint. Such a model was used for FE analysis of total ankle replacement during the stance phase of gait [32]. Contrarily, cuboid, and navicular were only broadly considered [33].

All of the works mentioned above did not employ the SSM analysis. Recently, a SSM for calcaneus has been described, where an accurate SSM of calcaneus was proposed [24]. The aim of this work was to extend that methodology to the case of the other three tarsal bones, namely cuboid, navicular, and talus. Additionally, it was of interest to ascertain whether SSM parametric characterisation can be used for classifying the particular tarsal bones.

## Methods

A method for automatically building a morphometric and anatomically accurate model of calcaneus was described in our previous work [24]. We follow that methodology aiming at developing SSMs for cuboid, navicular and talus. Retrospective volume data of 15 left and 15 right feet of male subjects were used. Scans were gathered from 30 different subjects. All subject records were anonymised and de-identified prior to processing according to the standard data release procedures. The Review Board of the Department of Radiology, Wroclaw Medical University, Wroclaw has approved the study. The study has been conducted according to the principles of the Declaration of Helsinki.

**Fig. 1:**
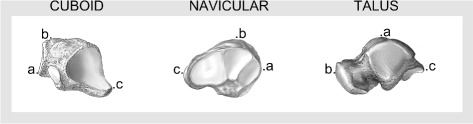
Anatomical landmarks for cuboid, navicular and talus Bone drawings adapted from [60]
  For cuboid: the lowest point of the surface for the fourth metatarsal (Point *a*_*C*_), the highest point of the surface for the fourth metatarsal (Point *b*_*C*_) and the most posterior point of the cuboid tuberosity which was the same as the lowest point of calcaneocuboid joint (Point *c*_*C*_).
  For navicular: the highest point on the superior edge of the navicular tuberosity (Point *a*_*N*_), the most posterior point of the navicular tuberosity articulating to medial cuneiform (point *b*_*N*_) and the most posterior point of the navicular tuberosity articulating to lateral cuneiform (point *c*_*N*_).
  For talus: the highest point of the trochlea (Point *a*_*T*_), the most posterior point of the head for navicular bone (Point *b*_*T*_) and the most posterior point of the posterior calcaneal articular surface (Point *c*_*T*_).
- **Averaging feature position and orientation:** Unification of models was prerequisite to further shape description. The subjects were scanned in the same feet-first, supine (FFS) position, but feet placement for each subject was slightly different. To unify the position of each bone the following steps are applied (see Fig. 2):
  1. Rotation of the model by an angle *α* between the plane *π*_0_:*z*=0 and the plane *π*_*abc*_ that includes points a, b, c of each considered bone.
  2. Translation of the model by the vector $\overrightarrow {C} = [x_{c}, y_{c}, z_{c}]$ to set the selected point (point *c* for all bones) in the origin.
  3. Rotation of the model about the *x* axis by the angle *β* which is between *x* axis and vector $\overrightarrow {A} = [x_{a}, y_{a}, z_{a}]$.

The particular steps of building the SSMs are: bones segmentation, land-marking, unifying feature position and orientation and SSM calculation. They consist of:

- **Image pre-processing:** The volume CT registered image is decomposed in order to prepare a series of 2D images in sagittal plane. For each 2D image the contrast is enhanced.
- **Contour extraction:** The region growing algorithm is applied to extract the contour [34]. A starting point was manually marked by an experienced operator.
- **3D point cloud to surface:** In order to obtain a surface from contours points the oriented normals are calculated. This is followed by Poisson surface reconstruction method for which the mesh is generated. Meshlab (Pisa, Italy) software was used to generate meshes [35, 36].
- **Land-marking:** For each bone, three anatomical landmarks were automatically marked on bone surface mesh. However, expert validation was still maintained to ensure that all points were correctly marked. In few cases an expert operator intervention was needed to correctly assign the points. The marked points are (see Fig. 1):

**Fig. 2:**
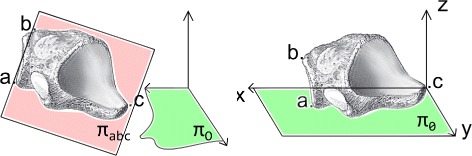
Averaging feature position and orientation. The illustration of the concept for averaging feature position and orientation, showed on cuboid example (bone drawings adapted from [60])
- **Spherical harmonics (SPHARM) decomposition:** The calculation of shape description is obtained by the SPHARM application [37–39].
- **Model and model order selection:** To estimate the optimal model order for SPHARM decomposition, the Minimum Distance Length (MDL) criterion [40] was used. The product of SPHARM decomposition, i.e., the set of coefficients estimated in the SPHARM expansion characterises the shape of bone. For the SSM descriptive statistics of SPHARM coefficients were calculated.

Two-way parametric ANOVA was used to test for changes between tarsal bones in SPHARM coefficients [41]. This was followed by an application of a machine learning technique, the Random Forest [42], to the 4-class recognition problem of tarsal bones. For that, the total of 120 samples of either left or right tarsal bone models (15 individuals × 4 bones × 2 left/right) were used. 10-fold cross-validation method [43] was used to assess the misclassification error. Sensitivity and specificity for each classified group was also calculated.

## Results

**Fig. 3:**
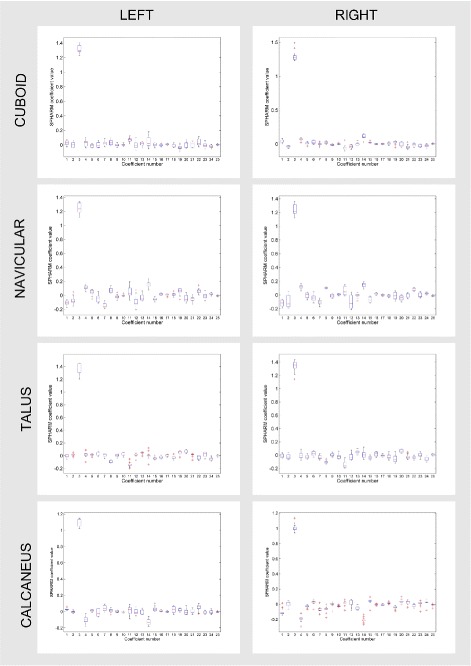
SPHARM estimates. The statistics of SPHARM estimates (*box-plots*) of the first 25 coefficients for the group of 15 left and 15 right models. Crosses indicate outliers

**Fig. 4:**
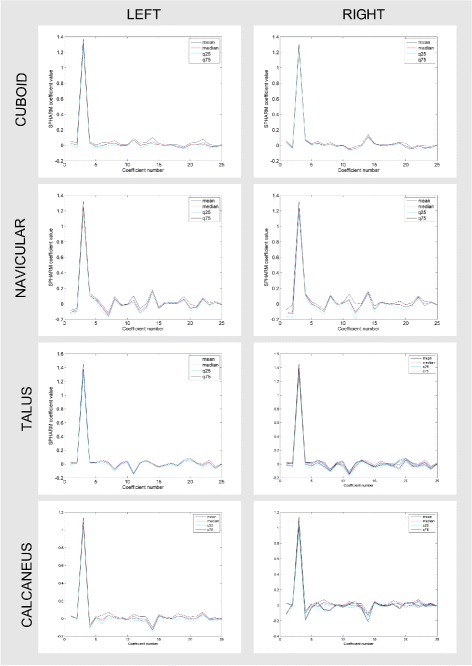
Descriptive statistics of SPHARM coefficients. The statistics of SPHARM estimates (the *first*, *second*, *third* quartile and mean) of the first 25 coefficients for the group of 15 *left* and 15 *right* models

**Fig. 5:**
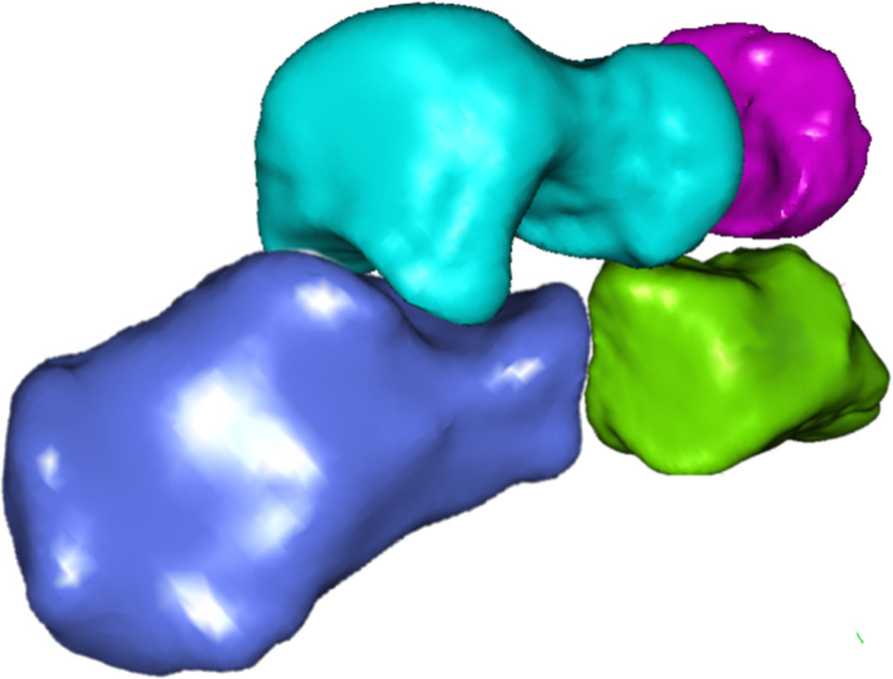
Reconstruction of models. An example of reconstructed SSM of cuboid, navicular, talus and calcaneus for the right foot (*blue* - calcaneus, *green* - cuboid, *magenta* - navicular, *cyan* -talus)

Figure 3 shows the box-plots for the first 25 SPHARM coefficients for left and right bones while Fig. 4 shows the first, second, and the third quartile as well as the mean of the 25 considered SPHARM coefficients. A 3D model of bones (see Fig. 5) is generated based on the mean estimate of coefficients for the right foot. Note that models shown in the figure are parted in order to exhibit the articular facets.

**Fig. 6:**
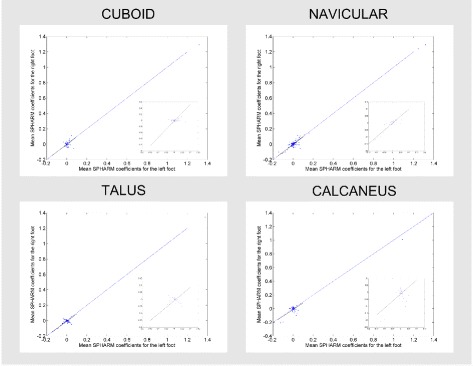
Correlation between the SPHARM coefficients. Correlation between the SPHARM coefficients of the left and the right models of cuboid, navicular, talus and calcaneus

Figure 6 presents the results of correlation between mean values of SPHARM coefficients for the right and left foot. The asymmetrical nature of bone can be assessed through examining the distribution of coefficients. The right/left foot correlation of estimated shapes is as follow: for cuboid (*r* ^2^=0.88), for navicular (*r* ^2^=0.99), for talus (*r* ^2^=0.98), for calcaneus (*r* ^2^=0.94), and statistically significant (*p*≪0.001) for all bones. Those correlations remain moderate when the highest coefficient is omitted, amounting to: for cuboid (*r* ^2^=0.72), for navicular (*r* ^2^=0.92), for talus (*r* ^2^=0.84), for calcaneus (*r* ^2^=0.54), and statistically significant (*p*≪0.001) for all bones (see Fig. 6 zoom).

Two-way ANOVA showed statistically significant differences between considered bones, coefficients, and interactions between the bones and coefficients (all *p*≪0.001). Two tests were considered. One for all coefficients and the other one in which the third SPHARM coefficient was excluded (see Fig. 3), as it was substantially greater than the other coefficients and could influence the test. Nevertheless, similar statistically significant results (all *p*≪0.001) were obtained for the reduced set of SPHARM coefficients. The distribution of SPHARM coefficients was found to uniquely characterise each bone and so this distribution could be used for bone classification.

Further, the random forest algorithm was applied to develop a tarsal bone classifier. Considering data cross validation, the optimal number of decision trees was 40 and for that the misclassification rate was 1.02%. Sensitivity and specificity was estimated: for calcaneus 0.9600 and 0.9953, for cuboid 0.9960 and 0.9878, for navicular 1 and 0.9996, and for talus 0.9793 and 0.9958, respectively.

## Discussion

Statistical shape modelling is a useful tool for feature extraction in medical imaging [12, 44]. The goal is to provide efficient information about the shape of an object of interest and its variability, often to build the so-called statistical atlas of particular body part, including bones [19, 45, 46]. Quantitative and accurate evaluation requires an appropriate representation used in shape modelling. The choice of the particular descriptors used in shape representation is important for further processing and analysis. The SPHARM description, used in this paper, provides quantitative information about the shape directly [47–49]. This paper contributes to this area by providing, for the first time, statistical anatomically accurate shape models for cuboid, navicular and talus.

Describing a shape using orthogonal polynomials, an inherent feature of SPHARM representation, allows for easy comparison of shapes through analysis of model coefficients. Further, it provides basis for classification of shapes based on testing for differences in the representative SPHARM coefficients. Using this methodology, our study shows that all considered tarsal bones can be uniquely represented by SPHARM.

Automated anatomical shape detection and classification have been considered in several applications of volumetric medical image analysis [32, 50–52]. Automated shape detection explores and applies the construction of algorithms that can learn from and make predictions on data. They are known as machine learning techniques and could assist in providing representative shape models as recently demonstrated by Cootes et al. [53], who used random forest regression voting for robust and accurate shape modelling. Among the many possible machine learning techniques we also employed the random forest algorithm but for the purpose of classification, which in our case showed high sensitivity and high specificity (both greater than 0.98) for all considered bones. The random forest technique is characterised by good accuracy for a relatively small number of samples (120 in our case) and containing a relatively high number of features (49 coefficients in the studied case). Also, it is robust to outliers in the input space and can rank the importance of variables considered in classification.

Another interesting aspect of statistical shape modelling is reconstruction [54–56]. The advantage of applying SPHARM to the shape reconstruction problem is their low complexity. Using estimated SPHARM coefficients it is possible to reconstruct one particular bone shape as well as create descriptive statistics for the examined group, say mean or median shape (see Fig. 5). It is worth noting that sexual dimorphism [57–59] was not considered in the study. The goal of the study was to develop anatomically accurate statistical models of tarsal bones and at that stage of research the size of bones was of concern. In other words, the statistical shape models of male bones are not necessarily scaled versions of their female equivalents.

## Conclusions

Summarising, the SSMs of cuboid, navicular and talus created in this work correspond to anatomically accurate morphometric atlases (SSM which includes morphological characteristics and provides mathematical representation of the shape) that have not been previously considered. They extend the considerable amount of 3D SSMs that are already employed in medical imaging. The new models of the considered tarsal bones are of interest in medical image analysis, orthopaedics and biomechanics and could provide additional information for automated identification of pathologies, better diagnostics and treatment, preoperative planning, as well as for implant design and procedures.

## Additional file

Additional file 1 SPHARM coefficients for calcaneus, cuboid, navicular and talus bones. (XLS 127 KB)
